# Cropland heterogeneity changes on the Northeast China Plain in the last three decades (1980s–2010s)

**DOI:** 10.7717/peerj.9835

**Published:** 2020-09-08

**Authors:** Xiaoxuan Liu, Le Yu, Qinghan Dong, Dailiang Peng, Wenbin Wu, Qiangyi Yu, Yuqi Cheng, Yidi Xu, Xiaomeng Huang, Zheng Zhou, Dong Wang, Lei Fang, Peng Gong

**Affiliations:** 1 Department of Earth System Science, Ministry of Education Key Laboratory for Earth System Modeling, Tsinghua University, Beijing, China; 2 Joint Center for Global Change Studies, Beijing, China; 3 Ministry of Education Ecological Field Station for East Asian Migratory Birds, Beijing, China; 4 Department of Remote Sensing Boeretang 200, Flemish Institute of Technology (VITO), Mol, Belgium; 5 Institute of Remote Sensing and Digital Earth,Chinese Academy of Sciences, Key Laboratory of Digital Earth Science, Beijing, China; 6 Ministry of Agriculture and Rural Affairs/Institute of Agricultural Resources and Regional Planning, Chinese Academy of Agricultural Sciences, Key Laboratory of Agricultural Remote Sensing (AGRIRS), Beijing, China; 7 National Supercomputing Center in Wuxi, Wuxi, China; 8 Chinese Academy Sciences, CAS Key Laboratory of Forest Ecology and Management, Institute of Applied Ecology, Shenyang, China

**Keywords:** Land-use change, Cropland area, Heterogeneity, Northeast China

## Abstract

The Northeast China Plain is one of the major grain-producing areas of China because of its fertile black soil and large fields adapted for agricultural machinery. It has experienced some land-use changes, such as urbanization, deforestation, and wetland reclamation in recent decades. A comprehensive understanding of these changes in terms of the total cropping land and its heterogeneity during this period is important for policymakers. In this study, we used a series of cropland products at the 30-m resolution for the period 1980–2015. The heterogeneity for dominant cropland decreased slowly over the three decades, especially for the large pieces of cropland, showing a general trend of increased cropland homogeneity. The spatial patterns of the averaged heterogeneity index were nearly the same, varying from 0.5 to 0.6, and the most heterogeneous areas were mainly located in some separate counties. Cropland expansion occurred across most of Northeast China, while cropland shrinking occurred only in the northern and eastern sections of Northeast China and around the capital cities, in the flat areas. Also, changes in land use away from cropland mainly occurred in areas with low elevation (50–200 m) and a gentle slope (less than 1 degree). The predominant changes in cropland were gross gain and homogeneity, occurring across most of the area except capital cities and boundary areas. Possible reasons for the total cropland heterogeneity changes were urbanization, restoration of cropland to forest, and some government land-use policies. Moreover, this study evaluates the effectiveness of cropland policies influencing in Northeast China.

## Introduction

The Northeast China Plain is one of the major grain-producing areas of China. During the past 30 years, the area has become the largest area of land cultivation activities in China, leading to remarkable changes to the natural landscape, as the cropland cover rapidly increased by 10% (Ye et al., 2009). Because of its fertile black soil and large fields adapted for agricultural machinery, the area is one of the main sources of production for soybeans and japonica rice (Frolking et al., 2002; Liu & Herbert, 2002; You et al., 2011), playing an important role in ensuring global food security (Shi et al., 2013). However, under the rapid reclamation of cropland for the natural environment, Northeast China has been facing a challenge between cropland expansion and the natural environment. Initially, excessive reclamation of grassland for farming, over cultivation, overgrazing, and deforestation can lead to land degradation and quality decline (Gao & Liu, 2010). Moreover, the cropland area and its grain production have also increased at the cost of wetlands shrinkage, affecting the sustainability of the ecosystem in this region (Wang et al., 2006).

Moreover, the continuous loss of agricultural land caused by urban growth in China is another issue of great concern for food security. With the progress of the Green Wall Policy (or Three-North Shelterbelt Reforestation program) and the Grain for Green Program (or Conversion of Cropland to Forest Program) in China, cropland in Northeast China converted to other natural land cover types under the policies (Piao et al., 2010). Thus, during the past several decades, cropland area has been subjected to large-scale land-use changes, such as urbanization, deforestation, and wetland reclamation (Zhao et al., 2019) and the amount with the spatial pattern of cropland is actually unclear. Therefore, a detailed analysis of cropland area change dynamics, especially of the evolution regarding the total cropping land area and land parcelling level, is important in spatially explicit crop modelling and crop yield estimation (Li et al., 2014; Sun et al., 2017b; Zhu & Woodcock, 2014).

For further cropland change analysis, many existing studies have considered the land cover mapping among the few past years as a baseline. Gong et al. (2013) and Yu, Wang & Gong (2013) studied land cover mapping at a global scale; however, they focused their attention only on a certain year. Yu et al. (2013) and Yu et al. (2014) also explored the cropland extent and conducted land cover mapping at multiple resolutions. Liu et al. (2005) and Liu et al. (2010) analysed more accurate land cover mapping in China. However, inadequate attention was paid to the long-time series in these studies. A yearly time series product since 2001 has been published by the MODIS Land Cover Type (MCD12Q1) (Friedl et al., 2010), but unfortunately it did not support change detection analysis (Cai et al., 2014). Bontemps et al. (2013) built a global long-term time series of earth observation data, which allows the derivation of transitions from one class to another in terms of the change in area (Li et al., 2018), but they were limited to resolution fineness and accuracy for local areas. Analysis at landscape scale would require the classification results at a much finer resolution (Yu et al., 2018); thus, high quality fine resolution land cover mapping with a long-time series in Northeast China is needed as a basis for the analysis of cropland changes.

Analyses were reported in previous studies to describe some attributes of cropland (White & Roy, 2015). Yan & Roy (2014) and Yan & Roy (2016) extracted crop field size in the United States from multi-temporal Landsat data (Yan & Roy, 2014; Yan & Roy, 2016) and Fritz et al. (2015) created a map for global cropland and field size (Fritz et al., 2015). These studies focused on the mapping method and fragmentation improvement, aiming at the field patch size. Yu et al. (2018) detected the changes in landscape patterns to portray cropland fragmentation. Fragmentation always leads to the heterogeneity of cropland, which is an important property that describes the variability of the observed surface properties in space (Chen et al., 2012). Because the cropland heterogeneity affects biodiversity or the environmental risks in crop fields (Martin et al., 2020; Sun et al., 2017a), it becomes a hot topic for researchers to focus on. There was some conclusion that different land uses with its heterogeneity could lead to a widespread decline in cropland biodiversity (Benton, Vickery & Wilson, 2003; Zhou et al., 2008). It was also found that the biodiversity in agricultural landscapes can be increased with the conversion of some production lands into more natural lands (Fahrig et al., 2011).

The heterogeneity of cropland is not conducive to the integrated management because of its petty and detailed distribution. Besides, heterogeneity is not good for crop development and growth under the invasion risks to heterogeneous environments (Goyal & Sharma, 2016). Crops are found as strongly dynamic objects and strong temporal changes in spatial heterogeneity are anticipated over cropland due to changes in fractional vegetation (Ding et al., 2014). Thus, the lack of cropland heterogeneity analysis should be paid attention to. Besides, most studies on field size or other cropland change characterization were limited in that the changes detected was restricted to a period between an initial state and an end state, or only in a particular year or a few separate years. Correspondingly, the exact point for the cropland change could not be detected in a certain year, which may lead to an ambiguous variation point when considering a longer period (Herfindal et al., 2012). In this study, we used a series of cropland parcelling products for the period 1980–2015 (Xu et al., 2020) and detected the variation of cropland heterogeneity, which represents the land-use status. The consistency analysis was carried out using statistical results of cropland size and the cropland heterogeneity. Besides, the possible reasons for cropland variation and heterogeneity change were also discussed. Finally, the fitness of the cropland area, heterogeneity changes, and policy implementation were explored.

## Materials & Methods

### Study area

**Figure 1:**
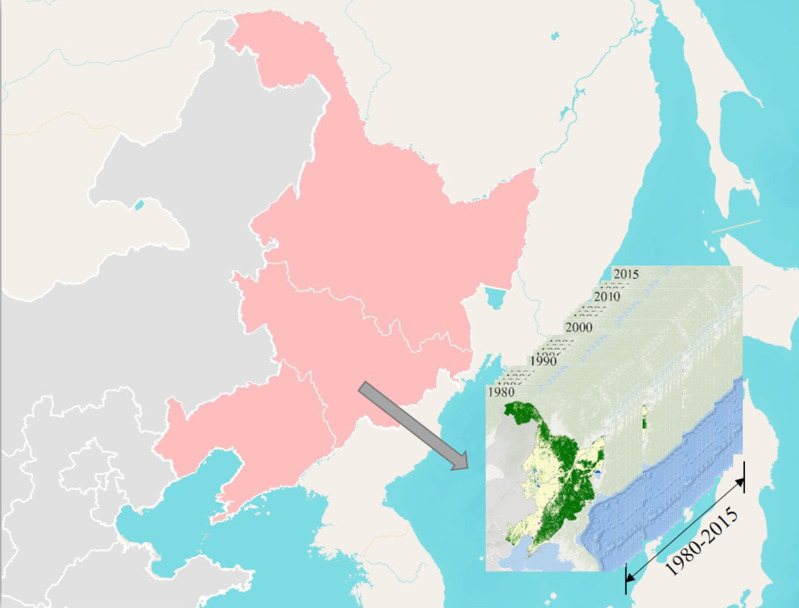
Study area and datasets. Land cover status (inset maps) of Northeast China based on classification results of Landsat data.

The Northeast China Plain is located in the northeast of China and mainly consists of Liaoning, Jilin, and Heilongjiang provinces (Fig. 1). Although the Northeast China Plain also encompasses the eastern part of Inner Mongolia, this study focused on the three above-mentioned northeast provinces. It contains many plains appropriate for cropping. The main plains, Songnen Plain, Sanjiang Plain, and Liaohe Plain are separately located in the northwest, northeast, and south of this area, and the region acts as a granary for the whole China. Around the plains are mountains, with the main vegetation type of temperate semi-humid forest and steppe in the east and temperate semi-arid steppe in the west. Considering most of the land policy was implemented in the county, we performed all the analyses on a county-level basis. The county level contains artificial landscape and natural landscape, and it is a middle scale landscape in China, like shire in England and county in American. And ecological studies of landscapes are linked closely with the administrative units (Fan & Ding, 2016).

### Land cover data

In this study, we used a set of continuous annual land cover mapping products at 30-m resolution (Xu et al., 2020), based on China’s Land-Use/cover Dataset (Liu et al., 2014b), which was generated from the Landsat Multispectral Scanner (MSS), Thematic Mapper (TM), Enhanced Thematic Mapper Plus (ETM +), Operational Land Imager (OLI), and multispectral data from the Huanjing-1 satellite (HJ-1). In the annual land cover mapping products, Xu et al. (2020) used multi-source remote sensing images including the Moderate Resolution Imaging Spectroradiometer (MODIS, 250 m) and the Advanced Very High Resolution Radiometer (AVHRR, 8 km) to derive original dataset. Normalized difference vegetation index (NDVI) data of the two remote sensing images were used via BFAST algorithm as a breakpoint detection analysis to identify the exact land cover change time in the annual land cover mapping products. The annual average accuracy for cropland, forest, and built-up land was 72.10%, 78.93%, and 91.89%, respectively. Figure 1 depicts the spatial distribution of each land cover class in Northeast China during 1980–2015. The relatively high spatial resolution and continuously mapping results make it possible to measure the annual cropland variation and the heterogeneity trend in Northeast China.

### Detection of cropland heterogeneity change

The cropland heterogeneity changes were detected using landscape diversity from 1980 to 2015, according to the Gibbs–Martin Diversity Index (Gibbs & Martin, 1962), to assess the overall utilization and status of land-use type. Also, there are some other indexes which are generally used in some research (Smith & Stanford, 1971; Spellerberg & Fedor, 2003), the Gibbs–Martin Diversity index is usually used to calculate diversification on crop diversification, appropriate for our cropland research (Rahaman & Singh, 2020). And many researchers using Gibbs–Martin Diversity Index for analysing diversification in recent years (Patel & Rawat, 2015; Rahaman & Singh, 2020; Sajjad & Prasad, 2014). We used the evolutive land cover diversity index as a measure of land heterogeneity and concentration, where a low index value can be seen as evidence of homogeneity and few land cover types (Gotham, Blum & Campanella, 2014). A high index value indicates heterogeneity and more land cover types distributed in a computing unit. The land cover diversity index is defined as:

\begin{eqnarray*}GM=1- \frac{\sum _{i=1}^{n}{f}_{i}^{2}}{{ \left( \sum _{i=1}^{n}{f}_{i} \right) }^{2}} \end{eqnarray*}

where *GM* represents the land use diversity index, *f*_*i*_ is the land cover area of type *i*, and *n* is the total number of types of land cover. In this paper, all indexes were detected at the county level, only considering cropland. To make sure the cropland area was nearly pure, over 50% in a 1-km cell was extracted to calculate the land cover diversity index county by county. A completely homogeneous cropland county would have a diversity index score of 0, while a perfectly heterogeneous county would have a diversity index score of 1 (assuming each land cover type in a county had the same area).

Changes in landscape patterns were assessed based on the cropland heterogeneity, including the spatial and temporal dimension, and used for cropland yearly variation analysis and heterogeneity analysis. For spatial analysis, the slope was calculated among all three decades, providing the annual variation of the heterogeneity situation. For temporal analysis, different sizes of cropland were analysed in turn to determine the pattern for different cropland sizes.

## Results

### Cropland heterogeneity variation

**Figure 2:**
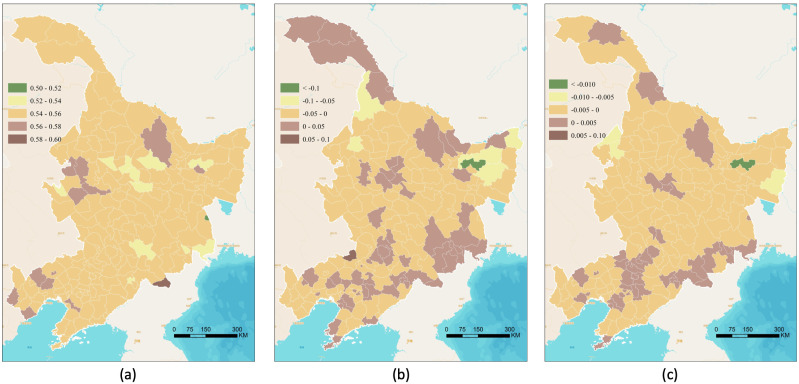
Heterogeneity analysis of cropland from 1980 to 2015 in Northeast China (unit: y^−1^). (A) The average of the heterogeneity index at county level, (B) bias of the heterogeneity index at county level, and (C) slope of the heterogeneity index at county level.

The distributions of the average, bias, and slope for the cropland heterogeneity index in Northeast China for the last three decades (1980–2015) at the county level are presented in Fig. 2, derived from the 30-m land cover mapping results. The spatial patterns of the averaged heterogeneity index (0.55 on average) were nearly the same, varying from 0.5 to 0.6, and the most heterogeneous areas were mainly located in some separate counties (Fig. 2A). The bias (Fig. 2B), which only considered the heterogeneity index in 1980 and 2015, has 122 counties in 182 (67% of the total counties) with the decreasing data, presenting a contracted pattern in most of the area and leading to an optimistic trend for the cropland concordance. Similar patterns could be seen in Fig. 2C, which represents the slope of the heterogeneity index at the county level. Counties of growth slope trends are 135 (74% of the total counties) and only 47 counties show a downward trend, indicating most areas are under homogeneity. What’s important, the slope covered all the years and gave an annual trend of the cropland heterogeneity variation, showing a closer fit with the cropland time series. Moreover, although cropland in most counties has become more homogeneous, the cropland heterogeneity still occurred in the east of Northeast China and some counties around the capital cities—Harbin, Changchun, and Shenyang. These areas are flatter are more favorable for urbanization, or probably change back to forest or other natural vegetation, responding to the Grain for Green Program.

### Cropland heterogeneity for 1980–2015

**Figure 3:**
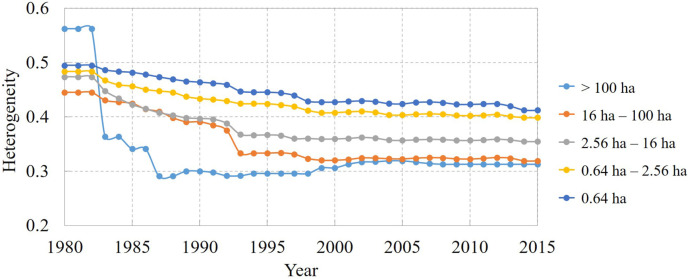
General trend of the cropland heterogeneity index for different cropland size levels in Northeast China from 1980 to 2015.

An elaborate way to describe cropland heterogeneity is to focus on the different sizes of cropland, which could help with distinguishing the heterogeneity influenced by the field size or land cover itself. Using a global field size dataset at 1-km resolution (Lesiv et al., 2019), we extracted all the cropland area over 50% in a 1-km cell, and then all the crop cells were considered in further statistical analyses. In this study, we used the annual heterogeneity index to describe the different cropland status variation of different cropland sizes from 1980 to 2015 (Fig. 3). In general, the smaller cropland size usually comes with a higher heterogeneity index because the smaller cropland is more easily combined with or superseded by other land cover types. Meanwhile, smaller cropland has less heterogeneity change and the heterogeneity index of cropland size within 0.64 ha changed just less than 0.1. Although the heterogeneity of the various cropland sizes was different among different periods, the trend for each cropland size decreased considerably during the past three decades, indicating increased cropland concentration. More strikingly, the largest cropland size over 100 ha changed substantially with a drop of more than 0.2 in the 1980s, representing the process for consolidating small plots of land into a bigger one.

With the separate time-series variation of the heterogeneity (Fig. 3), the cropland status development period could be divided into three sections. In the 1980s, the heterogeneity index of any cropland size decreased sharply, leading to the obvious increased homogeneity results in these years. In this period, cropland of the largest cropland size was homogenous firstly. Subsequently, between 1990 and 1995, large- and middle-sized cropland sizes were clearly homogenous, and other sizes of cropland were also homogenous because the heterogeneity index decreased gradually. The rapid progression of cropland homogeneity did not continue after 1995 (cropland heterogeneity decreased slowly for all field sizes) because the downward trend was considerably weakened after 2000. Overall, over the past 30 years, the cropland patch gradually became homogeneous because of the government policies of cropland consolidation.

### Cropland transfer patterns of elevation and slope

**Figure 4:**
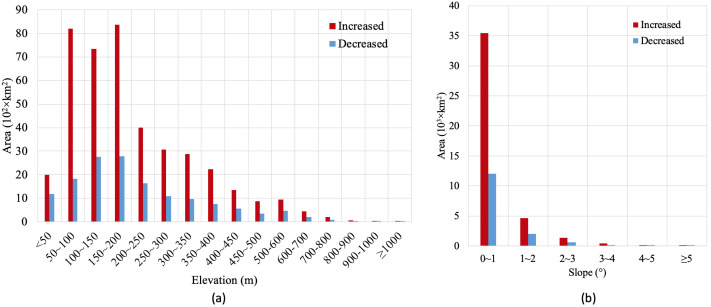
Cropland area changes. Gross cropland area changes in different (A) hierarchic elevation and (B) slope from 1980 to 2015.

Considering cropland change area of different elevations and slopes, areas with significantly increased cropland were mainly located at a higher elevation, expanding in areas at elevations between 50 and 200 m. The total increased cropland area at the elevation of fewer than 50 m and between 50 and 200 m is the most increased crop area, accounting for 5% and 57%, respectively (Fig. 4A). However, the decreased cropland proportion at the elevation of fewer than 50 m and between 50 and 200 m was 8% and 50% of all the decreased cropland area, respectively, indicating a decrease in cropland area at low altitude. For the cropland change along the slope, cropland expansion and contraction mainly occurred on slopes of less than 1° (Fig. 4B). In summary, cropland alteration mainly occurred in regions with low elevation and a gentle slope, because the cropland was always located on the smooth plain.

### Patterns combined total area and heterogeneity

**Figure 5:**
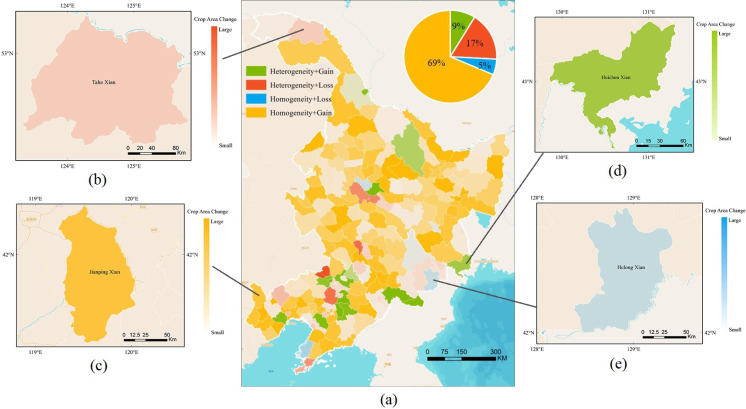
Four different patterns. (A) Counties and their proportions under the different patterns combining total area change and the cropland heterogeneity index time series. The four types are detected by different colours: (B) an example for cropland area gross loss and heterogeneity; (C) an example for cropland area gross gain and homogeneity; (D) an example for cropland area gross gain and heterogeneity; and (E) an example for cropland area gross loss and homogeneity. A more transparent colour indicates a smaller change range of total cropland area.

Figure 5 shows the four types (i.e., heterogeneity+gain, heterogeneity + loss, homogeneity + gain, homogeneity + loss) and their level for all the counties and their proportion with different patterns combining the total cropland area change and slope of the cropland heterogeneity from 1980 to 2015 in Northeast China. The variation of different combinations is shown in four colors: (1) cropland area gross gain and heterogeneity (16 counties, 9%); (2) cropland area gross loss and heterogeneity (31 counties, 17%); (3) cropland area gross loss and homogeneity (10 counties, 5%); and (4) cropland area gross gain and homogeneity (125 counties, 69%). Figure 5 shows that most of the counties gained cropland area in Northeast China (141 out of 182), and the cropland homogeneity occurred in most counties of Northeast China, (135 out of 182 counties), mainly located across most of the studied area, except capital cities and boundary areas.

In Northeast China, the predominant trend of cropland area gross gain and homogeneity represented nearly half of all counties, amounting up to 69%, but was scattered throughout Northeast China. Cropland expansion occurred in most of Northeast China, whereas cropland shrinking occurred only in the northern and eastern sections of Northeast China and around the capital cities in the flat areas. Furthermore, counties with cropland area gross loss and heterogeneity, occupying the second largest proportion of 17%, are mainly situated near the capital cities of the three provinces in Northeast China. The counties with cropland area gross gain and heterogeneity and the counties with cropland area gross loss and homogeneity showed a scattered distribution. The results suggest a strong trend of cropland area gross gain among the past 30 years and the cropland area showed little heterogeneity in most areas. However, trends of heterogeneity remain around the developing capital cities, corresponding to the urbanization process in China.

## Discussion

### The reliability of heterogeneity results

**Figure 6:**
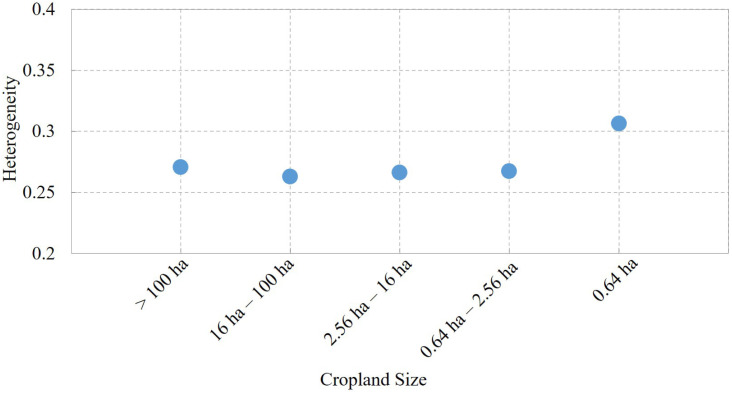
Consistency between mean heterogeneity results and cropland field size.

Although some research has used the heterogeneity index to analyse the complexity of the target (such as land cover type in the current study (He et al., 2010)), the reliability of heterogeneity for representing the complex cropland situation and the consistency between cropland heterogeneity and cropland field size still requires discussion. We calculated the mean heterogeneity index for each field size at the 1-km cell level (Fritz et al., 2015). As shown in Fig. 6, the smaller the cropland field size, the greater the heterogeneity index. This can be expected because the cropland with small field size is usually the smallholder farming area. Cropland of large field size, usually in ranches, is not less commonly transferred to other land cover types; thus, the larger the field size, the more homogeneous the cropland area.

However, it should be noted that the calculation results may be influenced by the land cover datasets and the accuracies of the land cover types may not be stable over the studied period (Congalton et al., 2014). Thus, the improvement for the reliability of the heterogeneity results may strongly rely on further progress in land cover mapping. However, based on this high-resolution land cover mapping of annual change (Xu et al., 2020), the exact year for the cropland change could be detected, and annual variation over the studied period was taken into account, ensuring the responsibility for cropland heterogeneity analysis over this long period.

### The reliability of results for cropland heterogeneity analysis

As China developed, an increasing number of policies were published that influenced the cropland status as Table 1 shows (Bryan et al., 2018; Song & Pijanowski, 2014). In the 1980s, with China’s rural land of the family contract responsibility system, and the land transfer system in transition, the adjustment of the land contract management rights increased cropland homogeneity. However, this became less apparent after 2000 since the cropland consolidation policy was implemented step by step, which can be detected in Fig. 3.

**Table 1:** Timeline of events influencing cropland in Northeast China.

**Year**	**Program**
1986	Land Administration Law
1987	Shelterbelt Development Program
1988	Comprehensive Agricultural Development Program
1989	Environmental Protection Law
1997	National Land Consolidation Program
1998	Natural Forest Conservation Program
1999	Grain for Green Program
2004	Decision on deepening reform and strict land management
2013	National Land Consolidation Plan
2015	Cultivated Land Quality Program

The results reveal considerable changes in cropland area gain and the homogeneity over the past three decades (Figs. 3 and 5). These cropland and heterogeneity variations could have many different causes. Figure 5 clearly reveals that the urbanization process of the Northeast China Plain is the main reason for cropland heterogeneity from indirect sources (Zhao et al., 2019). Besides, government land-use policies (e.g., the Grain for Green Program after 1999) also resulted in the cropland change (Liu et al., 2014a). The recent policies have worked out well for preventing forest damage, leading to slowed changes to heterogeneity in the 2000s.

Although the speed of total land consolidation is not especially rapid, most of the counties showed a trend of increasing homogeneity and concentration over the past three decades. Recently, an increasing number of policies have been implemented to protect the cropland and guarantee cropland safety. Although the speed of uptake is slow, cropland consolidation and homogeneity can be expected to improve over time.

## Conclusions

In this study, we presented comprehensive results of the cropland heterogeneity variation from a series of cropland products at the 30-m resolution for the period 1980–2015. The trend of total cropland heterogeneity has stabilized, with the decreasing trend having slowed down gradually over the three decades, especially in large pieces of cropland. Counties of growth slope trends are 135 (74% of the total counties) and only 47 counties show a downward trend, indicating the general trend for cropland concentration. Cropland expansion mainly occurred across Northeast China, while cropland shrinking coincidentally only occurred in the northern and eastern sections of Northeast China and around the capital cities in the flat areas. Also, changes in land use away from cropland mainly occurred in areas with low elevation (50–200 m) and a gentle slope (less than 1 degree). Although the cropland variation tendency varied among these three decades, the cropland showed gross gain and homogeneity, occupying 69% of all the counties. However, trends of heterogeneity remain around the developing capital cities, corresponding to the urbanization process in China. The possible reasons for cropland status variation include urbanization, restoring cropland to forest, and some government land-use policies, such as the Grain for Green Program.

##  Supplemental Information

10.7717/peerj.9835/supp-1 Supplemental Information 1 Raw Data: the results of different field size, and Figures 2, 4 & 5 Every group of the same name is a picture of XXX.tif, such as NEcounty.tif, Other files with the same name (NEcounty.tif.aux.xml, NEcounty.tif.ovr, NEcounty.tif.vat.dbf, NEcounty.tif.xml) are the attribute information of the tif file. It (XXX.tif) can be opened using GIS software such as ArcGIS. Other attribute files are automatically added in the software.

## Additional Information and Declarations

### Data Availability

The following information was supplied regarding data availability: The raw measurements are available as Supplemental File.
