# Supplementary figures and images for: Cropland heterogeneity changes on the Northeast China Plain in the last three decades (1980s–2010s)
Source: PeerJ. 2020 Sep 8;8:e9835. doi: 10.7717/peerj.9835 (PMC7485484; doi:10.7717/peerj.9835)

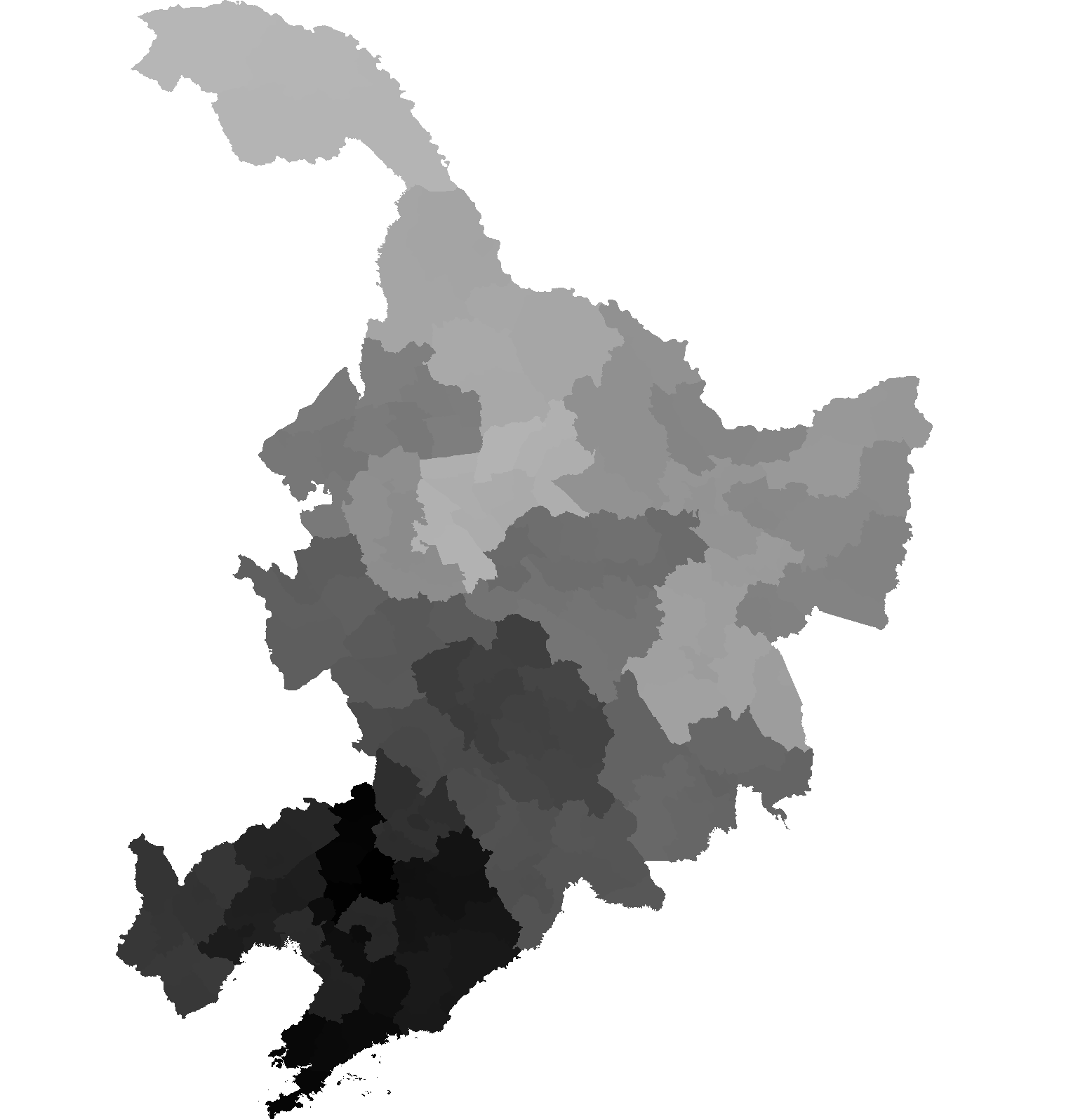

Supplement: Supplemental Information 1 — Every group of the same name is a picture of XXX.tif, such as NEcounty.tif, Other files with the same name (NEcounty.tif.aux.xml, NEcounty.tif.ovr, NEcounty.tif.vat.dbf, NEcounty.tif.xml) are the attribute information of the tif file. It (XXX.tif) can be opened using GIS software such as ArcGIS. Other attribute files are automatically added in the software. [file peerj-08-9835-s001.zip › CountyNew/NEcounty.tif]

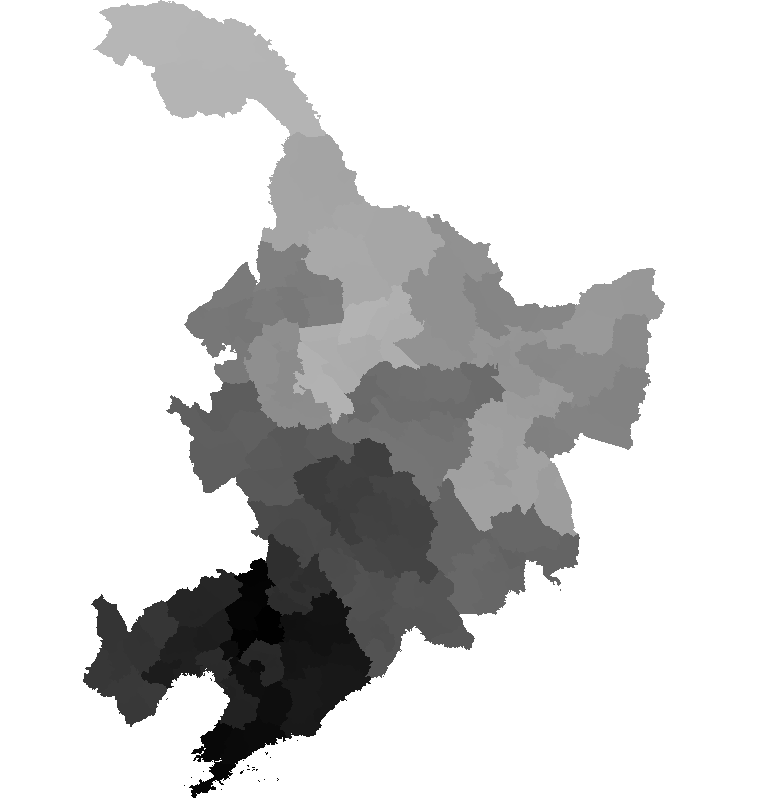

Supplement: Supplemental Information 1 — Every group of the same name is a picture of XXX.tif, such as NEcounty.tif, Other files with the same name (NEcounty.tif.aux.xml, NEcounty.tif.ovr, NEcounty.tif.vat.dbf, NEcounty.tif.xml) are the attribute information of the tif file. It (XXX.tif) can be opened using GIS software such as ArcGIS. Other attribute files are automatically added in the software. [file peerj-08-9835-s001.zip › CountyNew/NEcounty.tif.ovr]
